# Seeking Repeating Anthropogenic Seismic Sources: Implications for Seismic Velocity Monitoring at Fault Zones

**DOI:** 10.1029/2022JB024725

**Published:** 2022-12-30

**Authors:** Y. Sheng, A. Mordret, F. Brenguier, P. Boué, F. Vernon, T. Takeda, Y. Aoki, T. Taira, Y. Ben‐Zion

**Affiliations:** ^1^ University Grenoble Alpes University Savoie Mont Blanc CNRS IRD University Gustave Eiffel Grenoble France; ^2^ Institute of Geophysics and Planetary Physics University of California San Diego San Diego CA USA; ^3^ National Research Institute for Earth Science and Disaster Resilience Tsukuba Japan; ^4^ Earthquake Research Institute University of Tokyo Tokyo Japan; ^5^ Berkeley Seismological Laboratory University of California Berkeley Berkeley CA USA; ^6^ Department of Earth Sciences and Southern California Earthquake Center University of Southern California Los Angeles CA USA

**Keywords:** anthropogenic seismic sources, high‐frequency repeating signals, seismic velocity monitoring, seismic interferometry, stability of correlation functions

## Abstract

Seismic velocities in rocks are highly sensitive to changes in permanent deformation and fluid content. The temporal variation of seismic velocity during the preparation phase of earthquakes has been well documented in laboratories but rarely observed in nature. It has been recently found that some anthropogenic, high‐frequency (>1 Hz) seismic sources are powerful enough to generate body waves that travel down to a few kilometers and can be used to monitor fault zones at seismogenic depth. Anthropogenic seismic sources typically have fixed spatial distribution and provide new perspectives for velocity monitoring. In this work, we propose a systematic workflow to seek such powerful seismic sources in a rapid and straightforward manner. We tackle the problem from a statistical point of view, considering that persistent, powerful seismic sources yield highly coherent correlation functions (CFs) between pairs of seismic sensors. The algorithm is tested in California and Japan. Multiple sites close to fault zones show high‐frequency CFs stable for an extended period of time. These findings have great potential for monitoring fault zones, including the San Jacinto Fault and the Ridgecrest area in Southern California, Napa in Northern California, and faults in central Japan. However, extra steps, such as beamforming or polarization analysis, are required to determine the dominant seismic sources and study the source characteristics, which are crucial to interpreting the velocity monitoring results. Train tremors identified by the present approach have been successfully used for seismic velocity monitoring of the San Jacinto Fault in previous studies.

## Key Points

- Repeating anthropogenic seismic sources are useful for seismic velocity monitoring
- We use the stability of correlation functions over time as a proxy to search for persistent seismic sources
- Case studies in California and Japan demonstrate the potential for fault zone monitoring

## 1 Introduction

Fault zone activities, such as seismic ruptures, aseismic slips, and fluid migrations, affect seismic velocities (Brenguier et al., 2008; Peng & Ben‐Zion, 2006). Monitoring their temporal variations provides valuable insights into understanding the earthquake cycle. Despite the observations of velocity perturbation prior to laboratory earthquakes (Scuderi et al., 2016), very few studies from field experiments (Niu et al., 2008) have reported velocity changes as a precursor to large earthquakes. Active seismic velocity monitoring is hampered by the requirement of repeatable seismic sources, which is expensive for long‐term maintenance (Kumazawa & Takei, 1994). Instead, either natural (Ardhuin et al., 2011; Longuet‐Higgins, 1950) or anthropogenic activities (Lavoué et al., 2021; Riahi & Gerstoft, 2015) provide alternative sources of seismic waves that are suitable, though not by design, for monitoring purposes. Among different noise signals, microseism, generated from the interaction between oceans and the solid Earth, has been extensively used in the past decades. Microseisms are dominated by low‐frequency (0.1–1 Hz) surface waves and can be continuously recorded anywhere on Earth, making them accessible for various applications (e.g., Brenguier et al., 2008; Shapiro et al., 2005; Sheng et al., 2017). Since the pioneer application in Parkfield (Brenguier et al., 2008), seismic interferometry (Curtis et al., 2006) using microseisms has been successfully used to monitor post‐seismic relaxation following large earthquakes worldwide (e.g., Chen et al., 2010; Hobiger et al., 2012; Wegler et al., 2009). However, uncertainties about the microseismic sources (position, mechanism, frequency content) introduce estimation bias (Fichtner, 2015; Stehly & Boué, 2017; Tsai, 2009; Zhan et al., 2013), and careful examinations are often required to decipher between source and structure changes. In addition, microseisms are dominated by surface waves, which have a broad spatial sensitivity, posing large uncertainties in locating the velocity perturbation.

Human activities also generate powerful seismic waves that can travel tens of kilometers. For example, train‐generated seismic signals have been used for seismic velocity monitoring (Brenguier et al., 2019; Sheng et al., 2022) and inverting velocity structures at a shallow crustal scale (Pinzon‐Rincon et al., 2021). Car‐induced seismic waves have also been used to invert shallow velocity structures in either urban (Nakata et al., 2015) or rural (Meng et al., 2021) environments. Several characteristics make artificial seismic signals appealing for seismic velocity monitoring. Compared with microseisms, anthropogenic activities generate high‐frequency (>1 Hz) energy, including body waves that dive into the Earth and are sensitive to sharper velocity contrasts at depth. In addition, many of these opportune sources are fixed in space (e.g., windmills, drilling operations, cement factories) or operating following confined trajectories (e.g., trains and cars), implying a trackable source distribution. A fixed source is ideal for seismic velocity monitoring, as it allows to separate between changes associated with variable sources and those occurring in the medium.

Despite some successful applications (Pinzon‐Rincon et al., 2021; Sheng et al., 2022), opportune seismic sources have not been widely used. One basic challenge lies in identifying such sources from seismic data without prior knowledge. This study proposes a data‐driven workflow for finding persistent and powerful seismic sources that can potentially be used for velocity monitoring. Although our main target is monitoring fault zones, the workflow is also suitable for other purposes, such as the field of environmental seismology. The proposed algorithm builds upon seismic interferometry, which has been widely used as an effective method to extract coherent signals between seismic stations. The correlation functions (CFs) constructed from seismic interferometry have been treated as approximations of Green's functions between given station pairs. However, such an approximation requires a diffused wavefield (Lobkis & Weaver, 2001; Wapenaar & Fokkema, 2006), which is hardly satisfied in reality. Here, we treat CFs as self‐consistent observables (Sager et al., 2018), which carry the information about the seismic sources that generate the signals and the underlying structure through which seismic waves propagate. We further assume that medium perturbation has much less influence on the correlation wavefields than the temporal variations of seismic sources. This assumption is generally valid at the crustal scale, given the varying seismic source field (Zhan et al., 2013) and the relatively small (∼0.1%) velocity change resolved with CFs even after significant earthquakes (Brenguier et al., 2019). We note that analyses using autocorrelations resolve larger local co‐seismic velocity changes (e.g., Bonilla et al., 2019; Lu & Ben‐Zion, 2022), but the spatially averaged velocity changes affecting CFs are small. This study focuses on CFs and estimate their stability over time. The results can be used to detect strong, persistent anthropogenic sources, which repeatedly occur in time with a stable distribution in space.

## 2 Methods

### 2.1 Detecting Persistent Noise Sources

**Figure 1:**
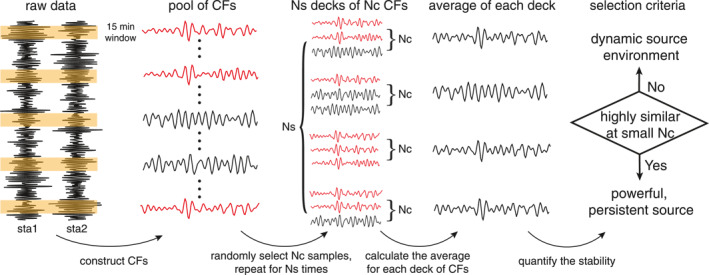
Workflow for detecting persistent, powerful seismic sources. Correlation functions (CFs) are constructed from continuous seismic data. Within the pool of CFs, some show high similarity to each other (marked by red) due to recurrent seismic sources. Bootstrapping is applied to estimate the stability of the CFs over time.

We focus on anthropogenic noise sources that last minutes to hours. A powerful and persistent seismic source contributes to highly coherent CFs among nearby stations. We treat the stability of CFs as a metric for source persistence. A random selection process is designed to estimate the stability of CFs, which are computed using cross‐coherence. Unless otherwise stated, vertical components are used for the analysis. Figure 1 illustrates the workflow schematically. For a given station pair, we first construct a pool of CFs, from which we randomly sample a certain number (noted as Nc) and compute their average. The selection process is repeated several times (marked as Ns) and results in a stack of averaged CFs (AvCFs). If persistent sources dominate the seismic wavefield, AvCFs from different attempts will be highly similar; however, if the seismic wavefield is highly variable, AvCFs will appear different. Therefore, the resemblance of AvCFs reflects the signature of the seismic source.

We use broadband stations in Southern California to illustrate the procedures. The goal is to search for high‐frequency seismic sources that can potentially be used for velocity monitoring at shallow seismogenic depths. We focus on the frequency band between 2 and 8 Hz, which appears to be a good compromise between relatively small wavelength (sharper sensitivity) and modest attenuation (Brenguier et al., 2019). Since the attenuation of such waves, we only examine station pairs within 30 km, significantly reducing the execution time. Data acquired in 2 months (July and August 2018) are analyzed. Previous studies (Brenguier et al., 2019; Pinzon‐Rincon et al., 2021; Sheng et al., 2022) have reported the retrieval of stable body‐wave CFs from seismic energy generated by freight trains in the Coachella Valley. The train tremors typically show clear energy for about 15 min on the seismograms. To test if we can successfully find train tremors using our methodology, we divide the continuous data into 15‐min‐long running time windows with 5 min overlap. Two months of data produce 8,640 CFs for each pair of stations, which are our starting pools of CFs.

**Figure 2:**
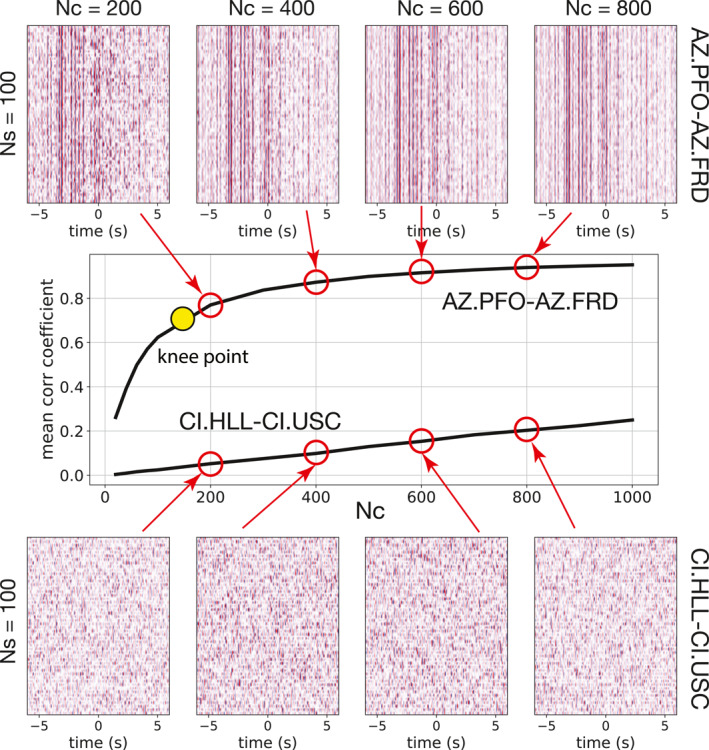
Examples for stability analysis of correlation functions (CFs). The top and bottom panels show the results for station pairs AZ.PFO‐AZ.FRD and CI.HLL‐CI.USC, respectively. The middle panel illustrates how the similarity among averaged CFs varies with increasing sample numbers. CFs for two station pairs have distinct behaviors.

We present two distinct examples in Figure 2: two stations in a remote area in the Anza region (AZ.PFO and AZ.FRD) and two stations deployed in the Los Angeles Metropolitan Area (CI.HLL and CI.USC). While AvCFs for station pair AZ.PFO‐AZ.FRD are very stable, even with few samples drawn from the pool, AvCFs for station pair CI.HLL‐CI.USC appear random. Correlation coefficients between every two AvCFs are calculated. Their mean value (MeanCC) is used as a proxy for the resemblance of the AvCFs. If Nc is unchanged, but Ns varies, MeanCC is relatively stable (Figure S1 in Supporting Information S1). Therefore, for the rest of the study, we keep Ns as 100 to reduce computational costs. If we fix Ns but increase Nc, MeanCC increases in both cases (Figure 2) but at varying rates. For station pair AZ.PFO‐AZ.FRD, MeanCC monotonically increases with Nc and achieves high values even when few CFs are sampled. For CI.HLL‐CI.USC, MeanCC increases gradually with Nc, and the values remain small even for large samples. The increasing trend is expected since larger Nc ensures more CFs are repeatedly sampled in different trials, leading to more similar AvCFs. We extract the coordinates of the knee point (the point of maximum curvature) from each curve (in the Nc vs. MeanCC domain) to quantify the MeanCC increasing rate. A fast rate implies specific seismic sources close to the station pair occurring very frequently, which is desired for our purpose. On the other hand, a slow rate suggests a variable environment with varying sources. Consequently, knee points with small Nc but large MeanCC values highlight the station pairs of interest.

**Figure 3:**
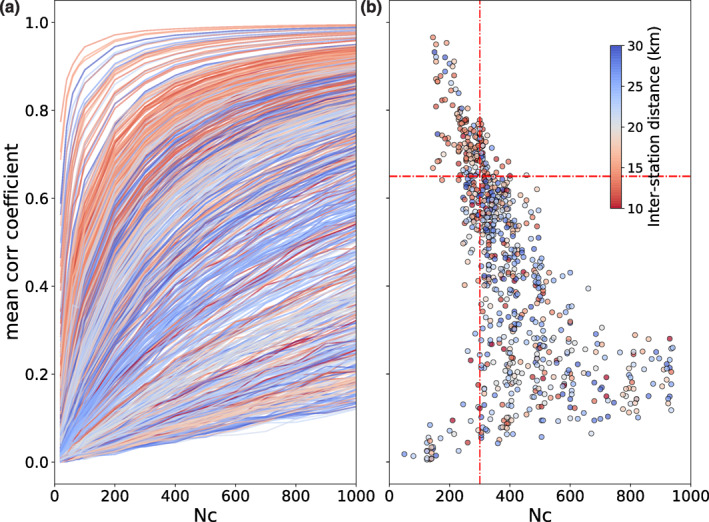
The correlation function (CF) stability analysis in Southern California. (a) Each curve is obtained from one station pair, showing the mean correlation coefficient varying with increasing samples of CFs. (b) The distribution of knee points taken from curves in (a). The inter‐station distances are colored and two dashed lines mark the selection criteria.

Figure 3 summarizes results based on the CF stability analyses for all station pairs in Southern California. Figures 3a and 3b show, respectively, the MeanCC for increasing sample sizes and the distribution of knee points. The latter are well clustered and form a linear trend at high MeanCC values but become scattered when MeanCC gets smaller. Generally, CFs of station pairs with shorter inter‐station spacing converge faster; however, very few station pairs that are over 20 km apart also show great promise. The former observation can be explained by wave scattering and attenuation during propagation, and the latter possibly indicates very powerful seismic sources.

**Figure 4:**
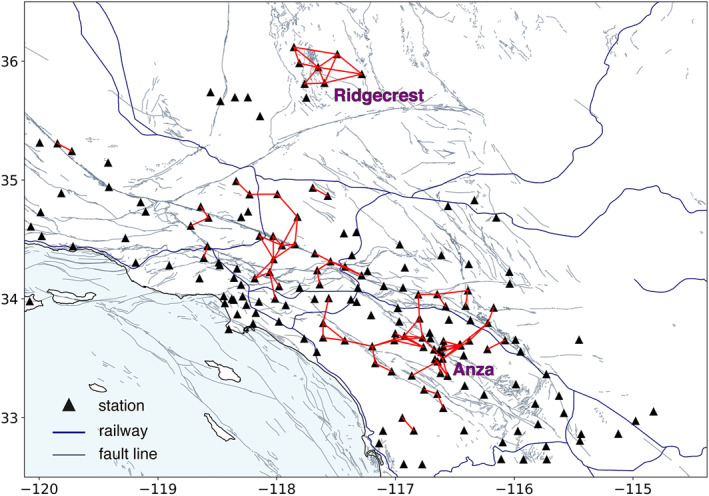
Map view of selected station pairs, connected by red links in Southern California. A dense cluster of red links implies the existence of powerful, persistent sources in the vicinity. Same plots with different selection criteria are given in the supplementary material.

We take Nc = 300 (equivalent to 75 hr of raw data correlated and stacked) as a threshold beyond which the measurements become quite scattered (Figure 3b). The threshold for MeanCC, which is chosen according to following the linear trend in the top left corner, is set as 0.65. Figure 4 maps the analysis results by connecting each station pair with a red line if the corresponding knee point falls in the top left corner in Figure 3b. The same results for different thresholds are shown in Figure S2 in Supporting Information S1. Both Anza and Ridgecrest areas show dense links among stations and are of great interest in fault‐zone seismic velocity monitoring. The Anza seismic gap, bounded by two seismically active fault segments while itself staying quiescent for about 220 years, has the potential to host an M 6+ earthquake in the approaching future (Rockwell et al., 2015). On the other hand, a sequence of M 6+ earthquakes struck Ridgecrest in 2019, producing significant stress changes on nearby faults and altering the regional seismic potential (Wang et al., 2020). Monitoring the spatial‐temporal variation of the stress state in both scenarios is crucial for earthquake hazard assessment.

The pattern shown in Figure 3 can be explained through theoretical analysis. For a given station pair, we want to compare two CFs x(t), y(t), each averaged from $N_{c}$ realizations. We write x(t) and y(t) as:

(1) $$x(t)=s(t)+n_{x}(t),$$

(2) $$y(t)=s(t)+n_{y}(t),$$

where s(t) is the common part between the two CFs, and $n_{x}(t)$, $n_{y}(t)$ represent the fluctuations that are uncorrelated with s(t). The correlation coefficient γ between x(t) and y(t) is a function of $N_{c}$ and could be written as:

(3) $$\gamma (N_{c})=\frac{\sum_{i=1}^{I}x_{i}y_{i}}{\sqrt{\sum_{i=1}^{I}x_{i}^{2}}\sqrt{\sum_{i=1}^{I}y_{i}^{2}}},$$

in which *I* is the length of the CFs. Plugging Equations 1 and 2 into Equation 3, we obtain:

(4) $$\gamma (N_{c})=\frac{\sum_{i=1}^{I}(s_{i}+n_{xi})(s_{i}+n_{yi})}{\sqrt{\sum_{i=1}^{I}{\left(s_{i}+n_{xi}\right)}^{2}}\sqrt{\sum_{i=1}^{I}{\left(s_{i}+n_{yi}\right)}^{2}}}\;.$$

**Figure 5:**
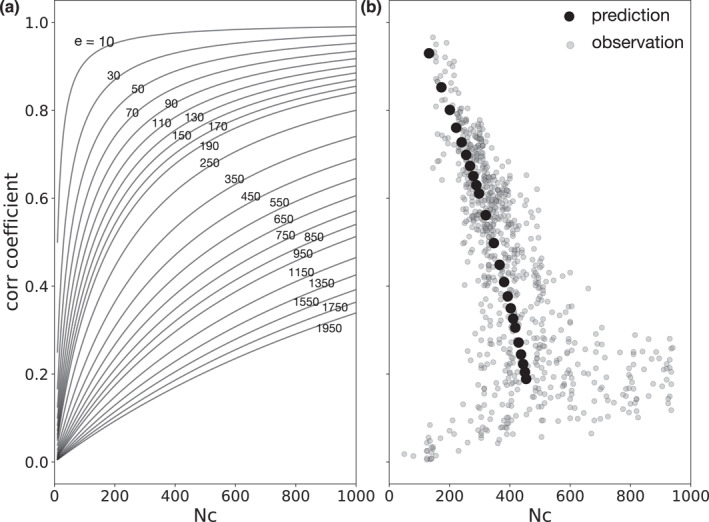
The correlation function stability analysis based on theoretical derivations. (a) The predicted correlation coefficient as a function of Nc for varying energy ratios between incoherent and coherent signals. The number on each curve gives the energy ratio e. (b) Knee points (black dots) obtained from curves in (a) compared with observations (gray dots) in Southern California.

Considering that s(t) is uncorrelated with $n_{x}(t)$ and $n_{y}(t)$, Equation 4 could be simplified as:

$$\gamma (N_{c})=\frac{\sum_{i=1}^{I}s_{i}^{2}}{\sqrt{\sum_{i=1}^{I}\left(s_{i}^{2}+n_{xi}^{2}\right)}\sqrt{\sum_{i=1}^{I}\left(s_{i}^{2}+n_{yi}^{2}\right)}}$$

$$=\frac{S^{2}}{\sqrt{\left(S^{2}+N^{2}\right)}\sqrt{\left(S^{2}+N^{2}\right)}}$$

$$=\frac{S^{2}}{S^{2}+N^{2}}$$

(5) $$=\frac{1}{1+{\left(\frac{N}{S}\right)}^{2}},$$

where $S^{2}$ and $N^{2}$ are the energy of s(t) and the fluctuations, respectively. Studies (e.g., Sabra et al., 2005) have shown that the convergence of CFs (the decay of the fluctuations) is proportional to the square root of $N_{c}$. Therefore, we have $N^{2}=N_{0}^{2}/N_{c}$, where $N_{0}^{2}$ represents the energy of the fluctuations on a single CF without stacking. With this, Equation 5 becomes:

$$\gamma (N_{c})=\frac{1}{1+{\left(\frac{N_{0}}{S}\right)}^{2}/N_{C}}$$

(6) $$=\frac{1}{1+\left(e/N_{c}\right)},$$

where $e={\left(\frac{N_{0}}{S}\right)}^{2}$ stands for the energy ratio between incoherent and coherent signals. Equation 6 describes the relationship between the correlation coefficient and $N_{c}$, while different station pairs are equivalent to using different energy ratios. Figure 5a illustrates how the correlation coefficient varies with $N_{c}$ given different energy ratios, and Figure 5b presents the corresponding knee‐point distributions compared with those obtained from the examined data sets. The theoretical prediction well captures the trend in the observation, indicating good consistency.

### 2.2 Identify Regional Sources

With a mixture of body waves and surface waves, the CFs from opportune high‐frequency sources are more complicated than those constructed from microseisms that are dominated by surface waves. A better understanding of the sources that contribute to the CFs is necessary for performing and interpreting seismic velocity monitoring. The stability of randomly selected CFs does not directly inform us of what or where are the generating sources. However, the one‐to‐one mapping between the CFs and the seismic data allows us to time the sources' occurrence. Different approaches can be applied to decipher the source signatures. We illustrate this idea using the station pair AZ.PFO‐AZ.FRD.

**Figure 6:**
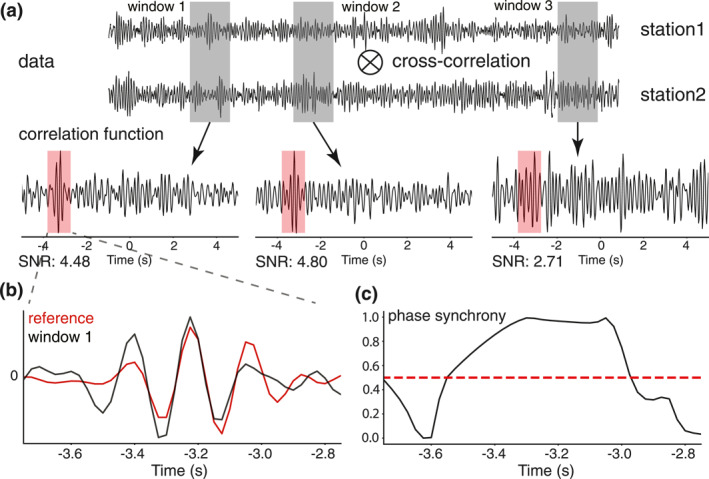
Illustration of finding correlation functions (CFs) containing a target phase. (a) Examples of CFs obtained from three different time segments. The first two satisfy the selection criteria of signal‐to‐noise ratio. The red box highlights the window of the target phase. (b) Comparison between the CF from window 1 and the reference, focusing on the target phase. (c) Phase synchrony measured from two traces in (b). The red line marks the level of 0.5.

The stacked CFs (Figure 2; Figure 6b) show a clear arrival around 3 s on the negative time lag, corresponding to waves propagating from station AZ.PFO to AZ.FRD. Considering the 18 km inter‐station distance, a phase arriving at 3 s has an apparent velocity of 6 km/s, consistent with the P‐wave velocity at mid‐crustal depth (Fang et al., 2016). A P‐wave arrival requires body‐wave sources occurring to the Northeast of station AZ.PFO to match with the direction of wave propagation. To confirm this hypothesis, we need to determine the corresponding sources and find the CFs that show significant arrivals at 3 s. We address this task using both phase and amplitude information. Figure 6 illustrates the procedure schematically. We use the average of all CFs as a reference and only focus on the anti‐causal part. The apparent arrival at 3 s is chosen as the target phase. We first reject uncorrelated CFs based on the signal‐to‐noise ratio (SNR), which is defined as the ratio between the peak amplitude of a 1‐s‐long time window surrounding the targeting seismic arrival and the standard deviation of the entire trace. CFs with SNRs smaller than three are excluded. We then use phase synchrony (PS; Rosenblum et al., 1996) to further select CFs. PS measures the phase resemblance between two traces at each time step. It is defined as:

$$PS=1-\sin\left(\frac{\left|a_{1}-a_{2}\right|}{2}\right),$$

where $a_{1}$ and $a_{2}$ are the instantaneous phases of the two traces estimated with the Hilbert transform. PS varies between 0 and 1, with larger values corresponding to greater phase consistency. Every CF is compared with the reference and is kept if the resulting PS value is larger than 0.5 for at least half of the time window.

**Figure 7:**
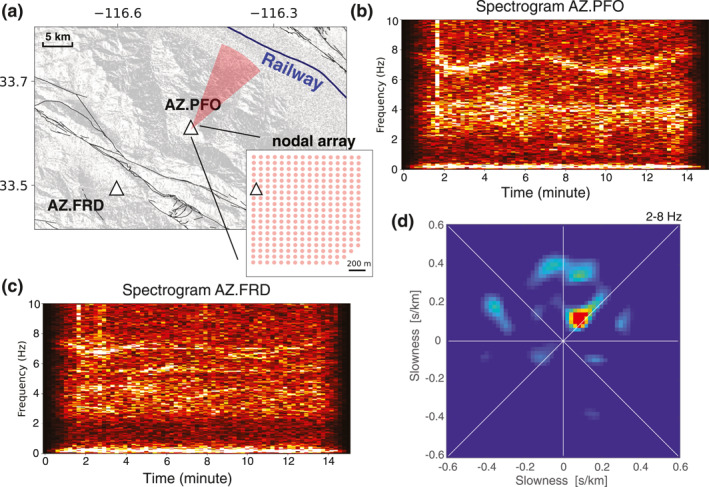
Investigating seismic sources in Anza. (a) Map of the seismic stations used. The nodal array is co‐located with permanent station AZ.PFO. (b) and (c) the example data spectrograms for station AZ.PFO and AZ.FRD, the same time segment as window 1 in Figure 6. (d) Beamforming diagram using the dense nodal array.

Once we have a collection of CFs, each with an emerging phase at around 3 s, we can trace back to the original seismic data and examine the source properties. One useful technique for locating sources is beamforming. A dense nodal array (network code 9K) was temporarily deployed at the Pinon Flat Observatory, co‐located with station AZ.PFO (Figure 7a). We perform beamforming analysis using the nodal array on a time segment selected based on previously discussed criteria. The result is presented in Figure 7d. The diagram exhibits a focal point to the Northeast side of the array, with a slowness of around 0.16 s/m, consistent with P‐wave velocity. Beamforming analysis confirms the proposed hypothesis that powerful sources generate P‐waves propagating from Northeast to Southwest. The spectrograms at both stations (Figures 7b and 7c) show an apparent oscillation between 6 and 8 Hz, possibly suggesting a moving source with varying speeds (Lavoué et al., 2021). A busy railway exists in the Coachella Valley to the Northeast of the Pinon Flat Observatory, and freight trains have been recognized as powerful seismic sources in the area (Brenguier et al., 2019; Pinzon‐Rincon et al., 2021). We, therefore, conclude that the apparent arrival on AZ.PFO‐AZ.FRD CFs is a P‐wave constructed from seismic energy generated by freight trains in the Coachella Valley. And station pairs like AZ.PFO‐AZ.FRD that cross the San Jacinto Fault provide a unique opportunity to monitor fault activities at seismogenic depths (Brenguier et al., 2019; Sheng et al., 2022).

## 3 Toward Generalization

**Figure 8:**
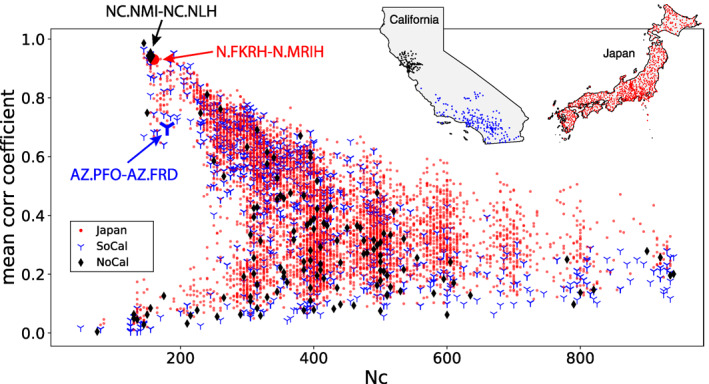
The distributions of knee points for three different data sets. The result for Southern California is the same as that in Figure 3b. Station pairs AZ.PFO‐AZ.FRD, NC.NMI‐NC.NLH and N.FKRH‐N.MRIH that are discussed as examples are highlighted.

We generalize the analysis to Northern California and Japan, both well‐equipped with seismic instruments for studying active tectonic processes. In Japan, High Sensitivity Seismograph Network (Hi‐net) stations are used, while in Northern California, data are acquired from the short‐period stations in the NC network. Two‐month continuous recordings are used for CF stability analysis in both cases, with September and October 2014 for Northern California and April and May 2012 for Japan. All other parameters are kept the same. Figure 8 plots the knee point distribution for the three different data sets, which all follow a similar trend. We thus keep the same selection criteria, that is, Nc < 300 and MeanCC > 0.65, to characterize stable station‐pairs CFs for the three seismic networks.

### 3.1 Japan

**Figure 9:**
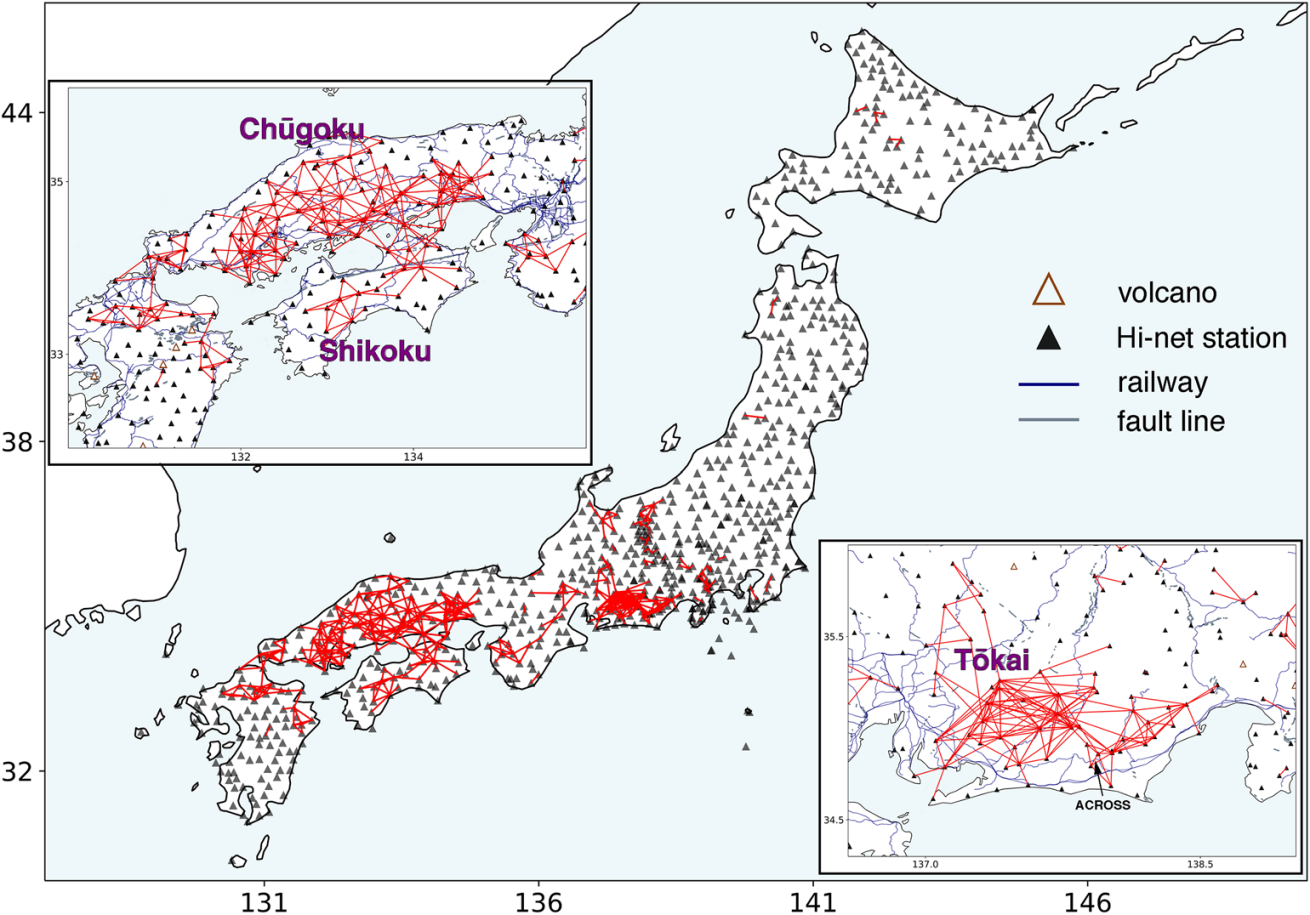
Map view of selected station pairs in Japan. The two insets highlight the regions with dense links.

The analysis in Japan is carried out on the entire Hi‐net network, and only the southern portions of Japan, including the Chūgoku Region, the Shikoku Island, and the Tōkai Region, show dense station links (Figure 9). Such a distribution indicates high consistency with the weights of cargo being transported by freight trains (Iwasa, 2001), suggesting that freight trains can also provide ample high‐frequency seismic energy in Japan. The distinction between the North and the South can also be affected by properties of the regional crust, as the Shikoku and Chugoku regions have weak crustal heterogeneity (Carcolé & Sato, 2010; Nishida et al., 2008) that favors coherent wavefields. Here, we focus on another type of source, the Accurately Controlled Routinely Operated Signal System (ACROSS), which generates vibrations by rotating its eccentric weights (Kumazawa & Takei, 1994). Frequency modulations are applied to the rotation, generating seismic signals in a particular frequency band. The high stability of ACROSS sources makes them ideal for monitoring temporal variations of seismic velocities (Saiga et al., 2006; Tsuji et al., 2018; Yamaoka et al., 2001, 2014).

**Figure 10:**
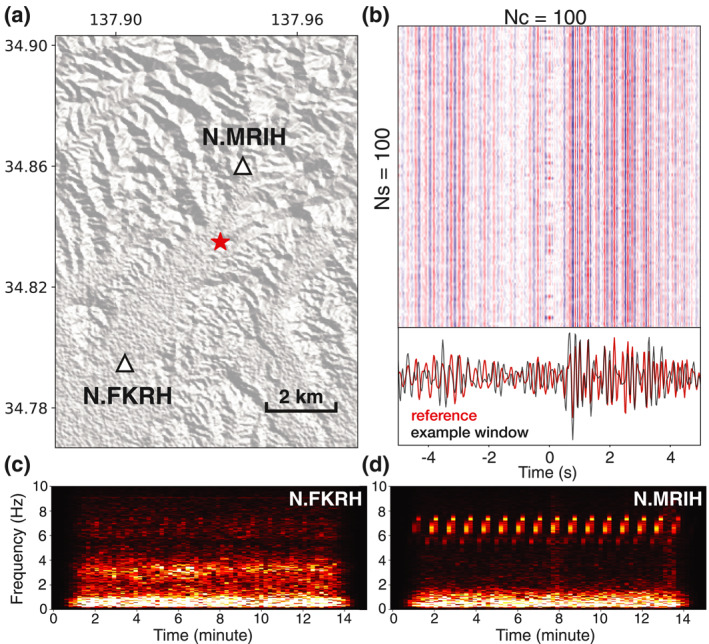
Investigating seismic sources in the Tōkai region with station pair N.FKRH and N.MRIH. (a) Map of the seismic stations used. The red star marks the Accurately Controlled Routinely Operated Signal System source. (b) The upper panel shows the average of randomly sampled 100 correlation functions (CFs). Clear signals emerge on the positive time lag. The lower panel shows the comparison between the reference and the CF obtained from an example time window. (c) The data spectrogram for station N.FKRH. (d) Same as (c) but for station N.MRIH.

One ACROSS source is of particular interest in our study. It is designed to operate at relatively low frequencies (3.5–7.5 Hz), with frequency modulation at an interval of 50 s (Tsuji et al., 2018). Such a frequency band overlaps with our frequency range of interest (2–8 Hz). The target ACROSS vibrates horizontally, generating strong S waves. It is deployed at Morimachi in the Tōkai region, where we observe a dense cluster of station pairs selected with our algorithm (Figure 9). To confirm that our workflow detects this ACROSS source, we examine the CFs for station pair N.FKRH‐N.MRIH, the two closest stations to the ACROSS source. The inter‐station distance between these two stations is around 8 km. A strong arrival at around 1 s on the positive time lag can be observed (Figure 10b). The relatively high apparent velocity (8 km/s) indicates a seismic source located between the two stations, closer to N.MRIH. The ACROSS source is 2.9 km away from station N.MRIH and 5.3 km away from N.FKRH (Figure 10a), consistent with the expected arrival time on the CF.

We find the occurrence times of the seismic sources responsible for the arrival at 1 s (Figure 10b) following the steps described in Figure 6. Figure 10 shows the spectrograms from an example time segment with the corresponding CF satisfying the selection criteria. On the spectrogram, especially for station N.MRIH, we observe a clear signal repeating every 50 s. Its energy dominates in the frequency range between 5 and 8 Hz, in excellent agreement with the characteristics of the ACROSS source. Successfully detecting the ACROSS source confirms the effectiveness of our algorithm. Interestingly, two other frequency bands, 2–4 Hz and 0–2 Hz show distinct signatures. Microseisms dominate the low frequency at both stations. The seismic energy between 2 and 4 Hz manifested at station N.FKRH but almost absent at N.MRIH is possibly from a local source. This highlights the complexity of seismic energy present in an urban setting.

### 3.2 Northern California

**Figure 11:**
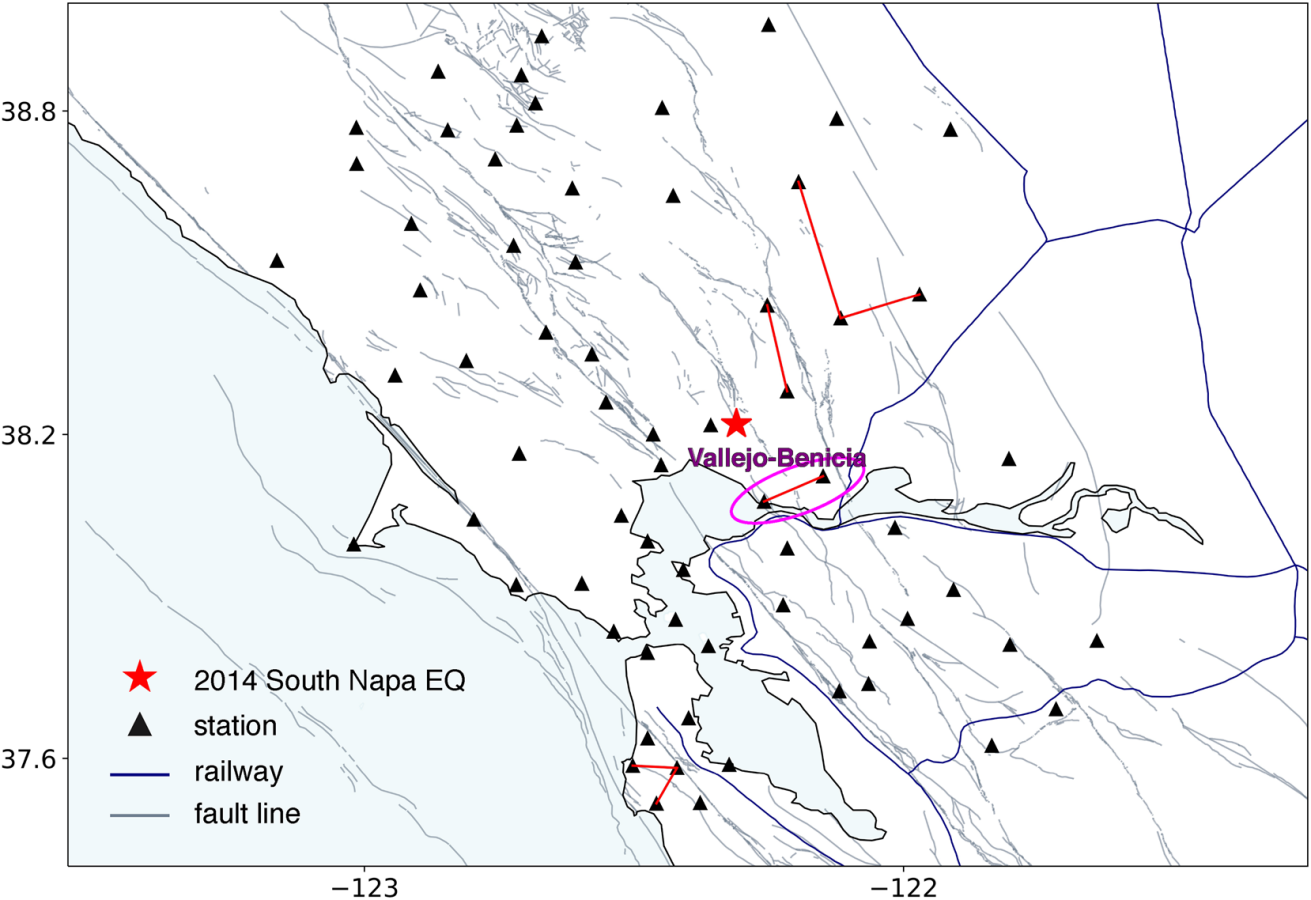
Map view of selected station pairs in Northern California. Station NC.NLH and NC.NMI are circled by a magenta ellipsoid.

**Figure 12:**
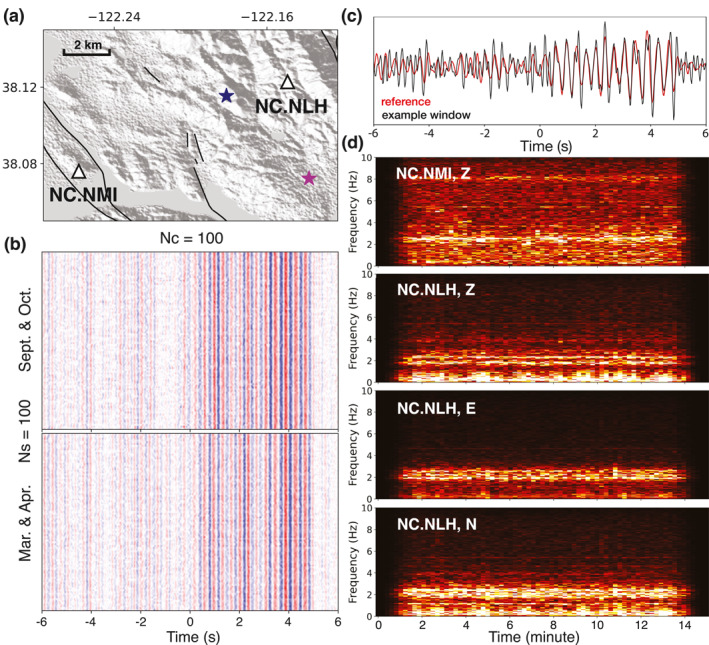
Investigating seismic sources in the Vallejo‐Benicia region with station pair NC.NLH and NC.NMI. (a) Map of the seismic stations used. The blue star marks the mining facility, and the purple star marks the oil refinery plant. (b) The average of randomly sampled correlation functions (CFs). Clear signals emerge on the positive time lag. The upper part shows the results for data acquired in September and October, after the 2014 South Napa Earthquake. The lower part shows the results for March and April, before the mainshock. (c) The comparison between the reference and the CF obtained from an example time window. (d) The data spectrograms. Horizontal spectrograms for station NC.NLH are also plotted. A band of energy between 2 and 3 Hz is observed on all channels.

Compared with the other two cases, fewer station pairs are selected in Northern California (Figure 11). Among the very few qualified candidates, station pair NC.NMI‐NC.NLH (11 km inter‐station distance) in the Vallejo‐Benicia region is of great interest. The close distances to the epicenter of the Mw6.0 24 August 2014 South Napa earthquake make this station pair a good candidate for studying earthquake‐induced seismic velocity change. However, stable CFs before and after the mainshock are necessary. In addition to the analysis performed on the data acquired in September and October, we repeat the same analysis in March and April 2014. AvCFs obtained from these two periods (Figure 12b) show remarkable phase consistency suggesting a specific seismic source acting persistently in the region.

The CFs exhibit a train of arrivals on the positive time lag (from NC.NLH to NC.NMI, Figure 12b), from 0 to 5 s. The non‐physical apparent velocities (∼11 km/s for the phase at 1 s) suggest the seismic source is located in between these two stations and is closer to NC.NLH. We focus on the phase between 0 and 2 s and search for the corresponding source. Figure 12d shows an example of seismic data that contributes to the target signal. The spectrograms exhibit clear energy between 2 and 3 Hz, especially for station NC.NLH. One mining facility is located to the West of station NC.NLH, and one oil refinery plant to its South (stars in Figure 12a). Industrial operations from both sites can potentially generate high‐frequency seismic energy, and their locations are both consistent with the arrival times between 0 and 5 s on the CFs.

**Figure 13:**
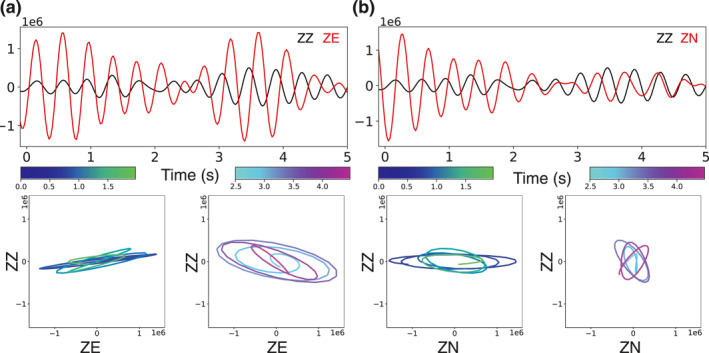
Polarization analysis using the correlation functions (CFs). (a) The analysis for vertical‐vertical (ZZ) and vertical‐east (ZE) CFs. The upper panel shows the waveforms, bandpass filtered between 2 and 3 Hz. The lower panels present the particle motions, color‐coded by time. (b) Same as (a) but for ZZ and vertical‐north (NZ) CFs.

Particle motion helps investigate the direction of arrival at a single station. If the seismic source is from the mining facility, East would be close to the radial direction for station NC.NLH. On the contrary, North would be the radial direction if the oil refinery plant mainly contributes to the seismic energy. Considering it is not straightforward to check polarization on the raw seismic data, we check it on the CFs. Since only the vertical component is available for station NC.NMI, in addition to the vertical‐vertical (ZZ) CF, we compute the CFs between two horizontal components on NC.NLH and the vertical component on NC.NMI, denoted as vertical‐east (ZE) and vertical‐north (ZN) CF. The CFs used in the polarization analysis are constructed using cross‐correlation without normalization in neither frequency nor time domain to preserve the relative amplitude information. The horizontal data spectrograms are shown in Figure 12d, and the particle motion analysis is shown in Figure 13.

The objective is to find which direction matches better with the radial direction, considering the particle motion of body‐ and Rayleigh waves. The CFs are filtered between 2 and 3 Hz to focus on the dominant seismic energy. We examine the particle motion for two wave packages. The ZE CF varies linearly with the ZZ CF during the first 2 s of the signal, and the polarization changes to elliptical motion from 2.5 to 4.5 s. If East is the radial direction, the first wave packet contains mainly body waves, and the second wave packet is the Rayleigh wave. On the other hand, the ZN CF peaks at the zero time lag, and the polarization is less pronounced. These observations suggest that the seismic energy is likely from the West, that is, the mining facility. A more conclusive interpretation, however, requires additional information.

## 4 Discussion and Conclusions

We design a workflow to search for powerful, persistent anthropogenic sources, aiming at extracting high‐frequency (>1 Hz) body‐wave CFs suitable for monitoring seismic velocity variations at a regional scale (∼10 km). Although our main motivation is to monitor fault zones, the workflow is well suited for other applications, such as monitoring permafrost degradation and CO_2_ sequestration. Our algorithm is based on estimating the stability of CFs over time and is tested on three different data sets to demonstrate its generality and effectiveness. We use the same set of parameters throughout this study, including a fixed 15‐min window length for cross‐correlation and the 2–8 Hz frequency band for CF stability analysis. These parameters can be adjusted according to data characteristics. The window length should be chosen such that the targeted seismic source dominates the CF. A 15‐min‐long time window is a good option in our case studies. It is selected according to the duration of train tremors but is also suitable for sources that last shorter but operate more continuously, such as the ACROSS source in Japan. The window length is not ideal for impulsive repeatable sources that occur only occasionally. Such sources are also valuable for investigating temporal changes in seismic velocities, but detecting them using the proposed algorithm requires much shorter time windows and involves extensive computation and possible memory overload. Other techniques, such as template matching, are more suited, assuming templates are already available.

We assume that typical seismic velocity changes have a minor effect on the correlation wavefields compared with the variations of the source distribution. We consider it a valid assumption in our application. Large earthquakes significantly alter the subsurface structure, and the post‐seismic local velocity variation can reach up to 10% or more (Bonilla et al., 2019; Lu & Ben‐Zion, 2022; Qin et al., 2020), but the spatially averaged co‐seismic velocity change is only on the order of 0.1% (Brenguier et al., 2008; Sheng et al., 2021). The more significant velocity changes that have been reported near the Earth's surface recover very shortly after the passage of the seismic waves. Such large perturbations over short durations are negligible for the time scale considered in this study.

We test our algorithm on data acquired from two consecutive months. Whether the results can be representative of other periods remains to be investigated. There are generally two strategies to pursue this endeavor. One is checking the CFs, which requires extensive computational resources, but is often straightforward to conduct. We use this approach in the Northern California case study, where we compare the CFs before and after the 2014 South Napa earthquake. Another option is directly searching for the seismic sources, assuming the source has been identified and its signature is clear. The case study in Anza presents a good example using train tremors. In this case, beamforming with a dense nodal array is particularly useful. It measures the direction of the seismic energy arrival and estimates the wave speed, which is critical for understanding the composition of the CFs.

Compared to only checking the CFs, determining seismic sources requires additional information, such as data from dense seismic arrays or operational schedules of nearby industrial activities. However, identifying the sources is necessary for conducting seismic velocity monitoring and further locating the velocity perturbation, especially for studies utilizing the ballistic arrivals instead of coda waves on the CFs. Anthropogenic sources typically present certain temporal variations, which affect the temporal resolution of velocity monitoring and require careful examination. Moreover, CFs can exhibit complicated waveforms, especially in the urban setting, due to the presence of multiple sources and the crosstalks between correlated sources (Ayala‐Garcia et al., 2021; Sheng et al., 2018). Recognizing and removing some sources of noise (Meng et al., 2019) can simplify the analysis of the remaining waveforms.

In addition to the proposed data‐driven techniques, numerical simulations of CFs (Sager et al., 2021) are beneficial to understanding the correlation wavefields. Anthropogenic sources often have specific spatio‐temporal distributions, which can be adequately simulated. In simulations, separating correlation wavefields excited by fast‐moving P‐waves from slow‐moving S‐ or surface waves is feasible. This provides guidance in explaining the observed wave packets and contributes to describing the sensitivity kernels for structural perturbations (Sager et al., 2021). Numerical simulations can also facilitate the study of CFs affected by source interactions, advancing a more comprehensive understanding of correlation wavefields.

## Supporting information

Supporting Information S1

## Data Availability

The seismic waveform data in California can be accessed through IRIS. The networks include the USGS Northern California Network (https://doi.org/10.7914/SN/NC), the ANZA Seismic Network (https://doi.org/10.7914/SN/AZ), the Southern California Seismic Network (https://doi.org/10.7914/SN/CI), the 2018 FaultScan dense array network (https://doi.org/10.7914/SN/9K_2018), and the Plate Boundary Observatory Borehole Seismic Network (no DOI available at present, but the information could be accessed through https://www.fdsn.org/networks/detail/PB/). The Japanese waveform data can be obtained through National Research Institute for Earth Science and Disaster Resilience, High Sensitivity Seismograph Network (https://doi.org/10.17598/NIED.0003).
