# Supplementary material for: Seeking Repeating Anthropogenic Seismic Sources: Implications for Seismic Velocity Monitoring at Fault Zones
Source: J Geophys Res Solid Earth. 2022 Dec 30;128(1):e2022JB024725. doi: 10.1029/2022JB024725 (PMC10078280; doi:10.1029/2022JB024725)
Supplement: Supplementary file 1 — Supporting Information S1 [file JGRB-128-0-s001.docx]

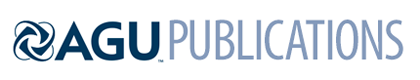


*JGR: Solid Earth*

Supporting Information for

**Seeking Repeating Anthropogenic Seismic Sources: Implications for Seismic Velocity Monitoring at Fault Zones**

Y. Sheng^1^, A. Mordret^1^, F. Brenguier^1^, P. Boué^1^, F. Vernon^2^, T. Takeda^3^, Y. Aoki^4^, T. Taira^5^ and Y. Ben-Zion^6^

^1^ Univ. Grenoble Alpes, Univ. Savoie Mont Blanc, CNRS, IRD, Univ. Gustave Eiffel, ISTerre, 38000 Grenoble, France

^2^ Institute of Geophysics and Planetary Physics, University of California, San Diego, La Jolla, CA, USA

^3^ National Research Institute for Earth Science and Disaster Resilience, Tsukuba, Japan

^4^ Earthquake Research Institute, University of Tokyo, Tokyo, Japan

^5^ Berkeley Seismological Laboratory, University of California, Berkeley, CA, USA

^6^ Department of Earth Sciences and Southern California Earthquake Center, University of Southern California, Los Angeles, CA, USA

**Contents of this file**

Figures S1 to S2

**Introduction**

This file contains two additional figures supporting the message delivered in the manuscript. Figure S1 shows how MeanCC varies with Ns. Figure S2 present the station pairs selected in Southern California with different criteria.

**Figure S1.** Mean correlation coefficient as a function of Ns. The analysis is performed on station pair AZ.PFO-AZ.FRD.


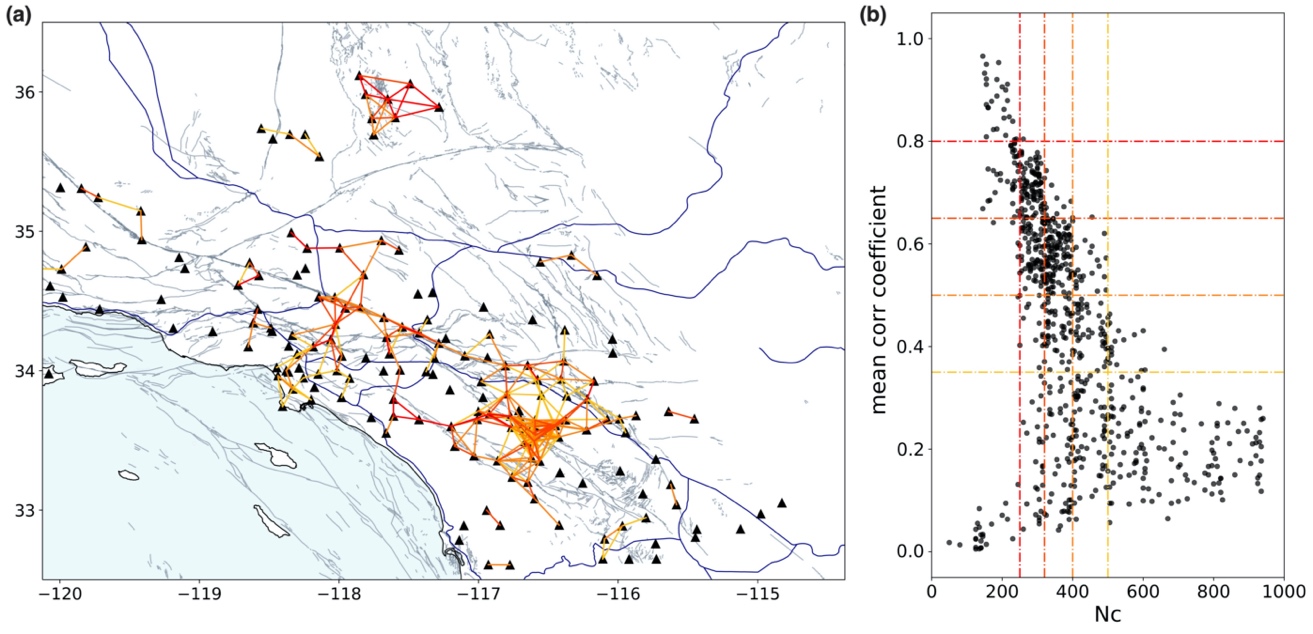


**Figure S2.** Selected station pairs in Southern California with different threshold colored by different colors. (a) map view of the selected station pairs. (b) knee point distribution, same as Figure 3b. The colored dashed lines mark different thresholds, each with the corresponding station pairs linked by the same color in (a).
